# 3D-Printed Poly(Lactic-co-Glycolic Acid) Binder-Based Self-Hardening Calcium Phosphate Bone Scaffolds

**DOI:** 10.3390/bioengineering13091049

**Published:** 2026-09-09

**Authors:** Savanah R. Sturm, Nicholas A. Mirsky, Adriana I. Sandino, Maria Castellon, Anshumi J. Desai, Isabella D. Guanche, Linh Johansson, Yago Raymond, Vasudev Vivekanand Nayak, Lukasz Witek, Paulo G. Coelho

**Affiliations:** 1 University of Miami Miller School of Medicine, Miami, FL 33136, USA; 2 Department of Oral Science and Translational Research, College of Dental Medicine, Nova Southeastern University, Fort Lauderdale, FL 33314, USA; 3 DeWitt Daughtry Family Department of Surgery, Division of Plastic Surgery, University of Miami Miller School of Medicine, Miami, FL 33136, USA; 4 Mimetis Biomaterials S.L., Carrer de Cartagena, 08025 Barcelona, Spain; 5 Department of Biochemistry and Molecular Biology, University of Miami Miller School of Medicine, Miami, FL 33136, USA; 6 Dr. John T. Macdonald Foundation Biomedical Nanotechnology Institute (BioNIUM), University of Miami, Miami, FL 33136, USA; 7 Biomaterials and Regenerative Biology Division, NYU College of Dentistry, New York, NY 10010, USA; 8 Department of Biomedical Engineering, NYU Tandon School of Engineering, Brooklyn, NY 11201, USA; 9 Hansjörg Wyss Department of Plastic Surgery, NYU Grossman School of Medicine, New York, NY 10016, USA; 10 Department of Oral and Maxillofacial Surgery, NYU College of Dentistry, New York, NY 10010, USA; 11 Sylvester Comprehensive Cancer Center, University of Miami Miller School of Medicine, Miami, FL 33136, USA

**Keywords:** hydrothermal processing, calcium phosphate, direct inkjet writing, 3D printing

## Abstract

Three-dimensionally (3D)-printed alpha-tricalcium phosphate (α-TCP) scaffolds, fabricated through a low-temperature hydrothermal dissolution-precipitation process, replicate the structural and compositional features of native bone. Reinforcing hydrothermally processed α-TCP with poly(lactic-co-glycolic acid) (PLGA) as a binder has previously been shown to confer distinct mechanical advantages, supporting its potential as a viable material for bone regenerative scaffolds. Although the hydrothermal processing of α-TCP scaffolds and PLGA-based mechanical reinforcement have each been characterized individually in earlier studies, this work represents the first pre-clinical in vivo assessment of osseoconduction and biocompatibility of 3D-printed, PLGA-reinforced, self-hardening calcium phosphate scaffolds in a large translational animal model. A ceramic ink suitable for extrusion was prepared by combining a 30 wt/vol% poloxamer 407 solution with α-TCP powder at a 0.45 wt/wt ratio (CTRL). A second extrudable ink, consisting of an α-TCP ceramic suspension incorporating a 35 wt/wt% PLGA binder, was formulated at a 0.5 wt/wt ratio (EXP). Cylindrical scaffolds (6 mm × 6 mm) were fabricated at room temperature using a custom-built Direct Ink Write 3D printer, then hydrothermally treated via submersion in water and thermal consolidation at 121 °C. Osteotomies were created in the ilium of adult sheep, with two cylindrical defects (7 mm × 6 mm) per animal, each receiving either a CTRL or EXP scaffold. Animals were euthanized at 3 and 12 weeks post-surgery (*n* = 6 animals per time point), and samples were collected en bloc for analysis. For both scaffold formulations, hard tissue formed by 12 weeks displayed high cellularity and active vascularization, consistent with early woven bone formation. Quantitative analysis revealed no significant between-group differences in bone formation at either time point (*p* > 0.05). These findings indicate that 3D-printed, PLGA-reinforced, self-hardening α-TCP scaffolds are osseoconductive and biocompatible, supporting their potential use in orthopedic and craniomaxillofacial bone defect repair.

## 1. Introduction

The synergistic integration between computer-aided design and advanced imaging tools such as microcomputed tomography (µCT) has allowed for the virtual reconstruction of hard tissue defect sites and their subsequent conversion into customized patient-specific scaffolds [1]. Additive manufacturing or three-dimensional (3D) printing of these customized scaffolds has enabled rapid bench-to-bedside translation owing to their low cost of production, a wide variety of materials available for use, and low turnaround times [2,3]. Of the various 3D printing modalities, the Direct Ink Write (DIW) technique, which originated with Cesarano et al. in 1998, has seen tremendous progress over the past decade and has been extensively used in the synthesis of bioceramic scaffolds [4]. The DIW technique utilizes a robotic gantry to sequentially deposit shear-thinning colloidal suspensions (inks) through a nozzle onto a substrate, layer-by-layer [4,5]. In contrast to alternative methods such as Fused Filament Fabrication, DIW presents superior speed and cost-efficiency (owing to the absence of the time-consuming filament production steps). This renders the entire process workflow of ink deposition (printing), drying, and sintering to conclude within a relatively short period.

Of the various Calcium Phosphate-based (CaP) ceramics that are utilized for bone regenerative procedures, hydroxyapatite (HA) and tricalcium phosphate (TCP) are the most common choices owing to their biocompatibility, favorable safety profile, and chemical resemblance to native bone [6,7]. Chemically, HA is stable and does not dissolve readily in biological fluids [8]. Although this chemical stability is necessary to preserve structural integrity, poor resorption kinetics (~1% annually) have been indicated to increase the potential risk of infection in vivo [9,10]. In contrast, β-TCP is fully resorbable in 6–18 months depending on the geometric size, shape of the scaffold, and its specific chemical configuration [11]. Moreover, time-dependent surface modification capabilities are an advantage; however, when heated to ~1125 °C, β-TCP transforms into α-TCP—a polymorph [12]. The added advantage of α-TCP over β-TCP has previously been attributed to its higher dissolution rates [12].

α-TCP-based bioactive bone cements have gained popularity as they can not only be molded with ease during operation but can also be subsequently implanted/injected into bone defects [13,14]. Another added advantage is its conversion to apatite after setting and hardening. This phenomenon of α-TCP hydrolysis was identified as the basis for the recent advancement of self-setting CaP inks. In brief, the hardening of α-TCP inks has been attributed to the cementitious reaction occurring in the ink at ambient temperatures, thereby eliminating the need for high-temperature sintering processes [15]. Furthermore, hydrolysis may also prevent volumetric changes associated with sintering ceramic constructs (densification), a well-known variable of ceramic processing [15,16]. Additionally, previous research has shown that calcium-deficient hydroxyapatite (CDHA), the reaction product of hydrolyzing α-TCP, comprises a similar chemical structure to that of native bone [17,18]. However, the aforementioned hydrolysis reaction is time-consuming relative to sintering, thereby restricting the use of this method in pre-clinical and clinical settings where rapid access to customized bone scaffolds may be necessary [16]. This drawback has been alleviated through the hydrothermal processing technique defined as controlled reactions occurring in the presence of aqueous solvents at high pressures/temperatures to dissolve and recrystallize materials that are relatively insoluble under standard temperature and pressure conditions [19].

Similarly, hybrid material strategies have been explored across additive manufacturing applications more specifically related to the polymer-reinforced ceramic approach. For example, the ability to include polymers in the composition of the self-hardening inks has been evaluated [20,21]. Polycaprolactone (PCL) has been used as a binder in a self-hardening α-TCP ink resulting in an increase in toughness of 3D-printed biomimetic apatite constructs [21]. However, while PCL has a promising reinforcing effect, its relatively low melting point has prevented it from being used in the hydrothermal processing technique [22,23]. As such, an alternative biocompatible and biodegradable aliphatic polyester, poly(lactic-co-glycolic acid) (PLGA), can withstand higher temperatures required for the hydrothermal process and improve the mechanical properties of 3D-printed scaffolds [24]. PLGA has also been shown to promote cell proliferation in vitro [24,25,26,27]. While the physicochemical and mechanical properties of PLGA-reinforced self-hardening α-TCP scaffolds have been established in a prior bench-top study [24], the in vivo osseoconductivity and biocompatibility have not previously been evaluated. Building on these findings, the current study aimed to evaluate whether 3D-printed PLGA binder-based self-hardening CaP bone scaffolds are osseoconductive and biocompatible for use in bone regenerative procedures in a large translational model.

## 2. Materials and Methods

### 2.1. Scaffold Synthesis

This study used cylindrical scaffolds (6 mm diameter × 6 mm height) in 2 formulations: a control (CTRL) scaffold corresponding to the hydrothermally processed condition previously reported by Raymond et al. [15], and an experimental (EXP) scaffold corresponding to the PLGA-reinforced (PLGA-B) condition previously reported by Johansson et al. [24]. In brief, CTRL (commercially available as MimetikOss^®^ 3D/creos™ syntoGain 3D, Mimetis Biomaterials S.L., Barcelona, Spain) was formulated by combining a 30 wt/vol% poloxamer 407 hydrogel with medical-grade α-TCP powder at a liquid-to-powder (L/P) ratio of 0.45 wt/wt [15]. For the EXP ink, a PLGA-based binder was first prepared by dissolving medical-grade PLGA (weight-average molecular weight: 179,650 ± 2256 g/mol, Corbion, Amsterdam, The Netherlands [24]) in 1,4-dioxane at 35 wt/wt% polymer concentration until a homogeneous, transparent solution was obtained. The medical-grade α-TCP powder was then incorporated into this binder at an L/P ratio of 0.5 wt/wt and mixed to achieve uniform ceramic particle dispersion [24]. A custom-made DIW 3D printer (Heavy Duty Paste Extruder, CIM-UPC, Barcelona, Spain) was used to synthesize scaffolds at ambient temperature [15,28]. The scaffolds were hydrothermally treated by submerging in water and thermally consolidating (at 121 °C in an autoclave at 1 atm) for 45 min, as per a previously established process workflow [15,24]. CTRL and EXP scaffolds were produced as a single batch per group. All samples were sterilized by autoclaving at 121 °C for 15 min. Exhaustive baseline physicochemical characterization of the CTRL and EXP scaffolds has been presented in prior studies by Raymond et al. [15] and Johansson et al. [24].

### 2.2. Surgical Procedure

Following approval from the Institutional Animal Care and Use Committee, *n *= 12 healthy and skeletally mature female sheep (24 months old) weighing approximately 65 kg underwent a 1-week acclimation period. All surgeries were performed under sterile conditions and general anesthesia, and as per the Animal Research: Reporting of In Vivo Experiments (ARRIVE) checklist presented in the Supplementary Materials. Anesthesia was induced with sodium pentothal (15–20 mg/kg) in Normasol solution administered via the jugular vein and maintained with isoflurane (1.5–3%) in a 50/50 O_2_/N_2_O mixture. Analgesia before and during the procedure consisted of subcutaneous sustained-release buprenorphine (0.20–0.27 mg/kg), together with intravenous morphine (0.12–0.24 mg/kg/hr), lidocaine (2–4 mg/kg/hr), and ketamine (0.36–0.72 mg/kg/hr). Vital signs were continuously monitored via ECG, SpO_2_, and end-tidal CO_2_. The ilium was selected as the surgical site for defect creation and scaffold implantation. The area was shaved and disinfected with iodine solution prior to osteotomy creation. After surgical preparation, a 10 cm anteroposterior incision was made over the iliac crest to expose the underlying bone, followed by creation of two 7 mm-diameter osteotomies per animal using subtractive drilling to a depth of 6 mm, performed under continuous saline irrigation.

Subjects were randomly allocated to one of two healing times, 3 or 12 weeks (*n* = 6 animals per time point), and each osteotomy received either a CTRL or EXP scaffold (*n* = 1 scaffold per group per animal). Assignment of CTRL versus EXP scaffold to the medial or lateral osteotomy site was performed for each animal, without a pre-specified randomization sequence. The order in which the two defect sites were prepared and treated with the scaffolds was not alternated across animals. The defect was intentionally sized slightly larger than the scaffold to enable atraumatic scaffold placement and to avoid excessive stress on the peripheral bone walls that would result from press-fitting. The same gap was consistently achieved in all specimens. Scaffold stability was maintained through layered soft tissue closure, with muscle sutured using Vicryl 2-0, and skin closed using nylon 2-0. To reduce complications, cefazolin (500 mg) was administered pre- and post-operatively. Animals were provided food and water ad libitum for the healing periods. Post-operative analgesia was administered via subcutaneous injection of buprenorphine sustained release (0.20–0.27 mg/kg) every 3 days, as needed. Animals were euthanized (3 and 12 weeks following the surgical intervention) and regions of interest, encompassing the scaffold and surrounding bone/soft tissue, were harvested en bloc. Only study surgeons (LW and PGC) were aware of the group distributions throughout the course of the study.

### 2.3. µCT and Volumetric Reconstruction

At the study endpoints, the collected samples were thoroughly cleaned by sharp dissection. Following the removal of excess soft tissue, the samples were immersed in a 10% neutral buffered formalin solution for 48 h. Subsequently, they were transferred to 70% ethanol and subjected to µCT scanning. The samples were scanned (µCT 40, Scanco Medical, Basserdorf, Germany) with a voxel resolution of 18 µm, at 70 kVp and 114 μA. The calibration and phantom data are provided in the Supplementary Materials. Volumetric reconstruction, quantification, and visualization were performed on data imported into Amira v6.3.2 software (Thermo Fisher Scientific, Waltham, MA, USA) in digital imaging and communication in medicine (DICOM) format. Scaffold and bone were distinguished based on their respective Hounsfield Units (HU; bone: 3512–5850, scaffold: 5850–32,767 in 16-bit DICOM image stacks) by a single trained and calibrated user, who was blinded to treatment group and time point. Bone (%) and scaffold (%) in this analysis are expressed as the percentage of the total defect volume occupied by bone and scaffold material, respectively. As such, it should not be interpreted as a direct volumetric measure—such as from a fixed baseline mass or volume (especially for scaffold (%)).

### 2.4. Histological Processing and Analysis

After µCT imaging, samples underwent dehydration through a graded ethanol series (70% to 100%) prior to infiltration and embedding in methacrylate-based resin. Embedded blocks were sectioned into 300 μm-thick slices using a low-speed wafering saw (Isomet 2000, Buehler Ltd., Lake Bluff, IL, USA), and each section was mounted onto acrylic slides with a cyanoacrylate adhesive (Loctite 408, Henkel AG & Co. KGaA, Düsseldorf, Germany). Slides were then ground down to a final thickness of 100 μm using a grinding system (Metaserv 3000, Buehler Ltd., Lake Bluff, IL, USA) with progressively finer silicon carbide abrasive papers under continuous water irrigation, followed by 1 min of polishing with a micro-fiber cloth and an alumina-based polishing suspension (MicroPolish™ Alumina, 1 μm, Buehler Ltd., Lake Bluff, IL, USA). After thorough rinsing, sections were stained with Stevenel’s blue and Van Gieson's picrofuchsin and imaged using an automated slide scanner (Aperio CS2, Leica Biosystems, Deer Park, IL, USA).

### 2.5. Quantitative Histomorphometric Analysis

Stained histological sections (1 central section per scaffold per animal) were evaluated to quantify the bone, scaffold, and soft tissue present within the defect. As with the volumetric µCT analysis, bone (%), scaffold (%), and soft tissue (%) in this analysis represent the proportion of the total defect area occupied by each respective tissue/material type. Bone, scaffold, and soft tissue were manually identified and separated into different “layers” within image editing software (Photoshop 2024, Adobe Systems Incorporated, San Jose, CA, USA) by a single trained, experienced histopathologist blinded to treatment group and time point. The layers were then each assigned different colors [soft tissue in red (RGB value = 255, 0, 0), bone in green (RGB value = 0, 255, 0), and scaffold in yellow (RGB value = 255, 255, 0)] and merged into a Tagged Image File Format (TIFF) and quantified using proprietary image analysis software (JV Analysis 2014, NYU Dentistry Biomaterials and Regenerative Biology Division, New York, NY, USA/University of Miami Miller School of Medicine Department of Biochemistry and Molecular Biology, Miami, FL, USA).

### 2.6. Sample Size and Statistical Analysis

The sample size was established *a priori* by power analysis, where at least 24 scaffolds (6 scaffolds per group per time point) were necessary to attain 80% power at a type I error rate (α) of 0.05 and an effect size of 0.7 (G*Power, v3.1.9.7, Heinrich-Heine-Universität Düsseldorf, Düsseldorf, Germany), with bone (%) from µCT volumetric reconstruction serving as the primary outcome variable. Data obtained from volumetric reconstruction and histomorphometry were analyzed using a general mixed model with scaffold group as a fixed factor and animal identity as a random intercept in IBM SPSS (v29, IBM Corp., Armonk, NY, USA), with results expressed as means and corresponding 95% confidence intervals (95% CI) and statistical significance set at *p* < 0.05. Osteotomy position was not included as a covariate, and pairwise comparisons were conducted using least significant difference post hoc analyses.

## 3. Results

Gross evaluation of the surgical sites did not reveal signs of infection, inflammation, or other adverse clinical effects. Additionally, upon harvest and dissection, there were no major indications of scaffold fractures.

### 3.1. Qualitative Histological Findings

**Figure 1:**
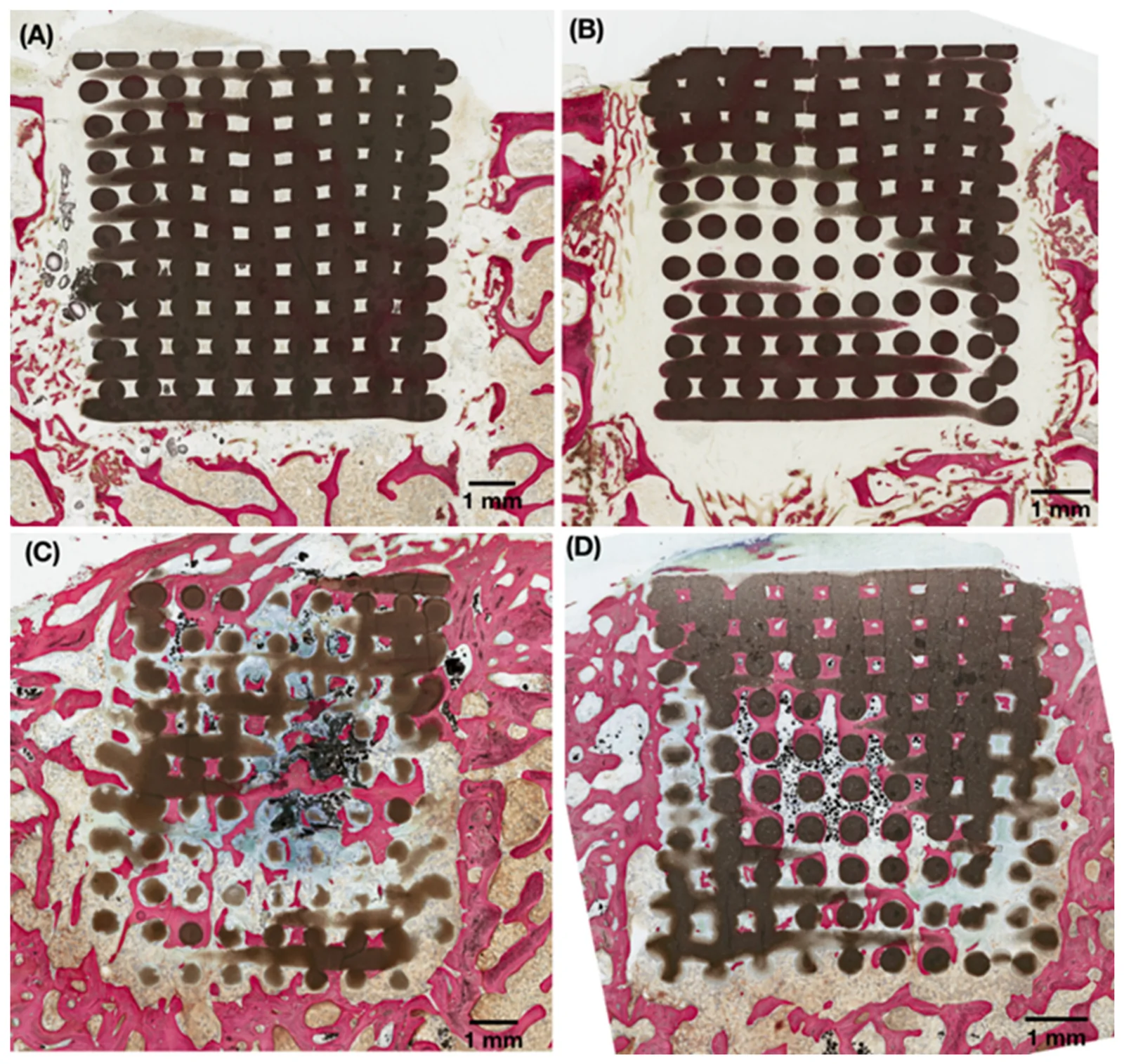
Representative low-magnification histomicrographs of the (**A**,**C**) CTRL scaffold group after 3 and 12 weeks in vivo, respectively, and (**B**,**D**) EXP scaffold group after 3 and 12 weeks in vivo, respectively. Extracellular structures and cells are colored in blue/blue-green, collagen fibers are colored in green-blue, bone is colored in red, osteoid material in yellow-green, muscle fibers in blue or blue-green, and the scaffold in black.

**Figure 2:**
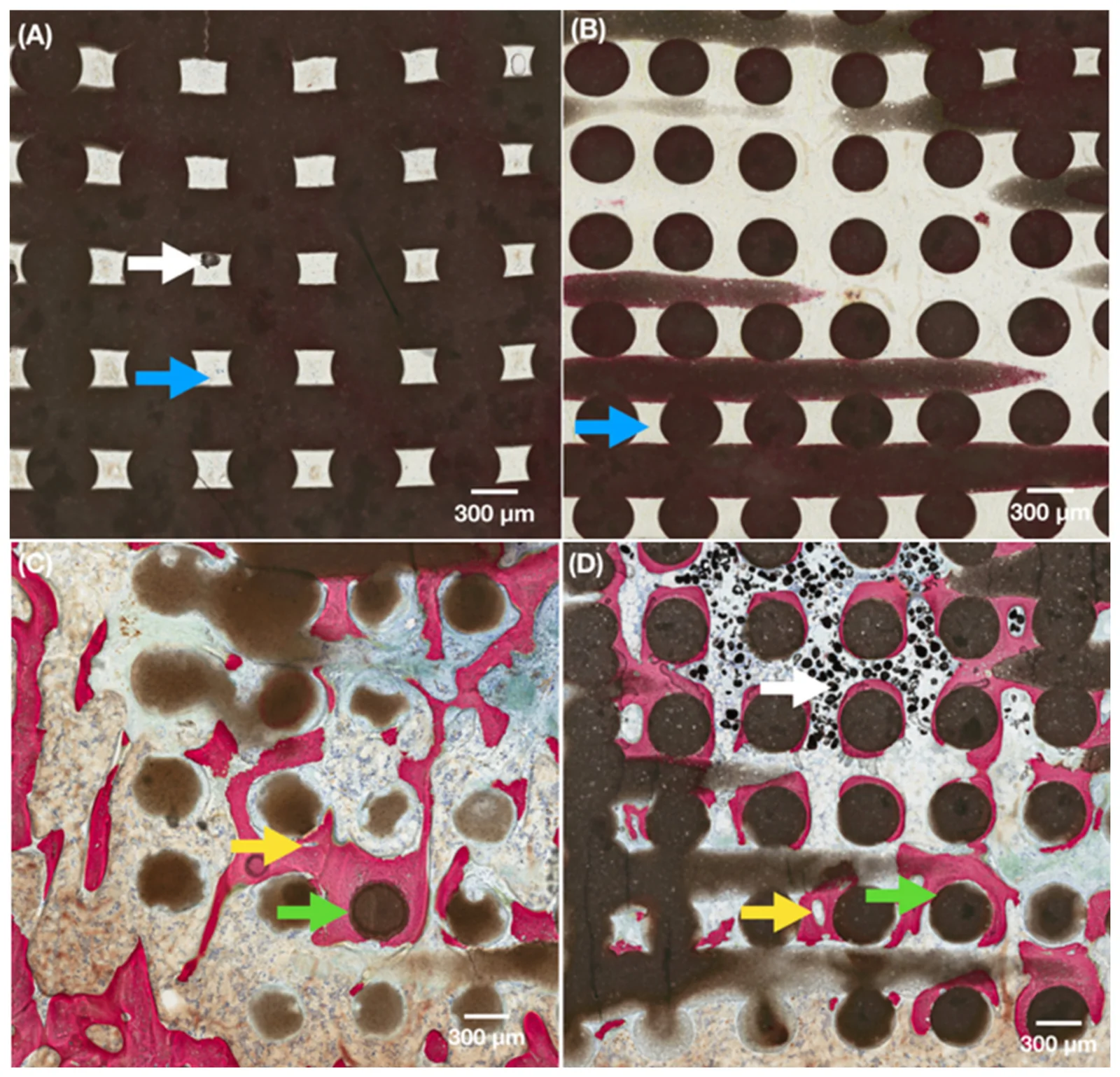
Representative high-magnification histomicrographs of the (**A**,**C**) CTRL scaffold group after 3 and 12 weeks in vivo, respectively, and (**B**,**D**) EXP scaffold group after 3 and 12 weeks in vivo, respectively. Blue arrows point toward the inner engineered pore structure of the lattice-like scaffolds, white arrows point toward embedding-related artifacts, green arrows highlight intimate contact between bone and the scaffold surface, and yellow arrows depict bone remodeling sites.

At 3 weeks, bone regeneration was limited to the immediate walls of the osteotomy, with the inner lattice-like structure of the scaffolds largely devoid of hard tissue presence (Figure 1A,B). In contrast, non-decalcified histologic sections revealed extensive bone formation in both groups at 12 weeks (Figure 1C,D). Compared to the earlier healing time point (Figure 2A,B), bone formation at 12 weeks extended throughout the full length and diameter of the scaffold lattice in both groups, with osseoconduction evident along the construct surfaces (Figure 2C,D, green arrows). In both scaffold formulations, the newly formed hard tissue showed high cellularity and vascularization, consistent with early woven bone deposited via an intramembranous-like pathway. Additionally, some areas within the scaffold pores in both groups showed remodeling (Figure 2C,D, yellow arrows). Of note, qualitatively, increased bone formation was accompanied by a decreased presence of soft tissue—primarily comprising loose connective tissue.

### 3.2. µCT and Volumetric Reconstruction

**Figure 3:**
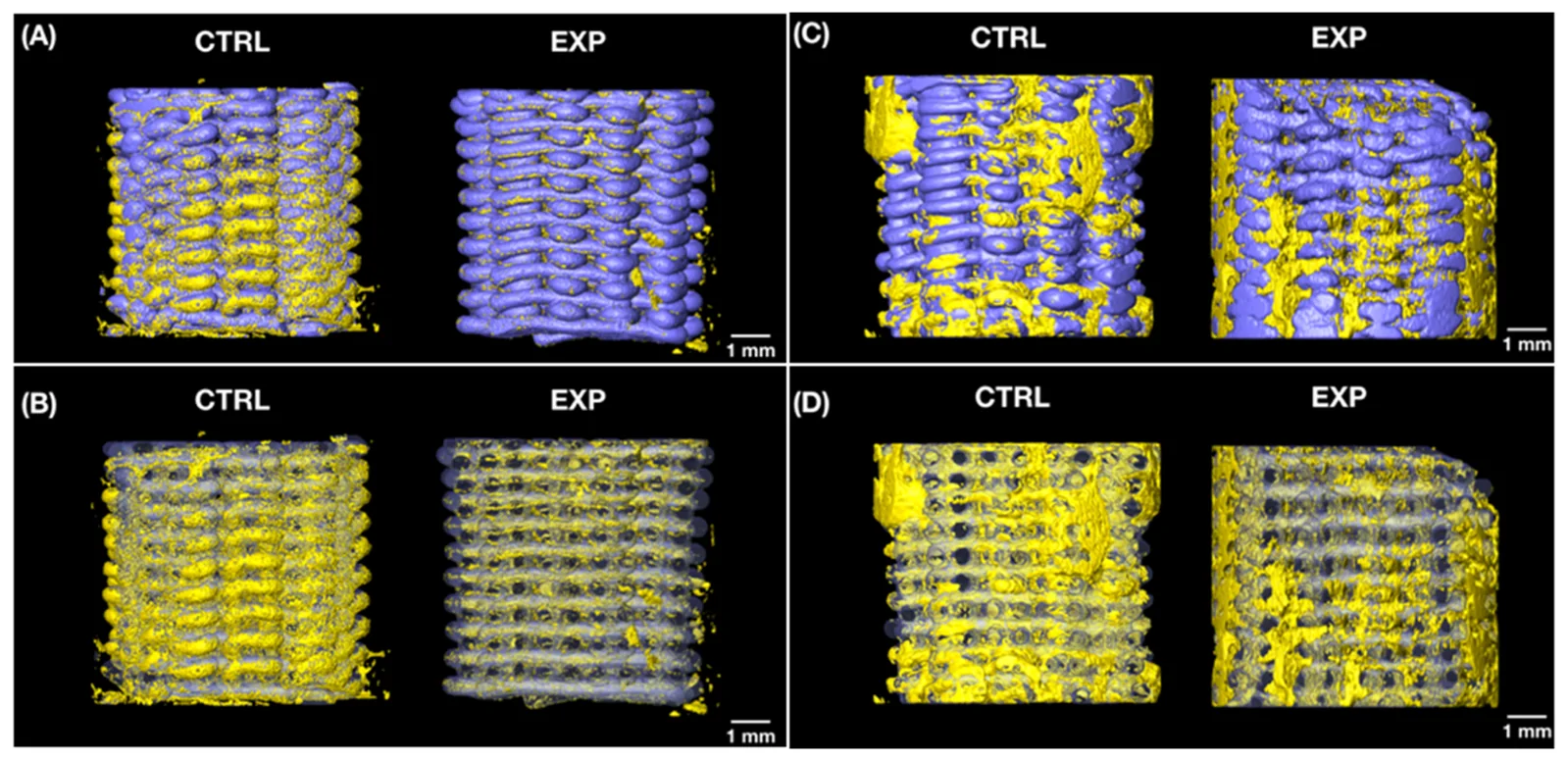
Volumetric reconstruction of the scaffolds placed within the osteotomies after (**A**,**B**) 3 and (**C**,**D**) 12 weeks of permitted healing. Yellow = Bone, Purple = Scaffold. (**A**,**C**) depict bone and scaffold layers in full opacity (100%); (**B**,**D**) demonstrate the same region of interest with the opacity parameter of the scaffold layer (purple) set to 50%.

**Figure 4:**
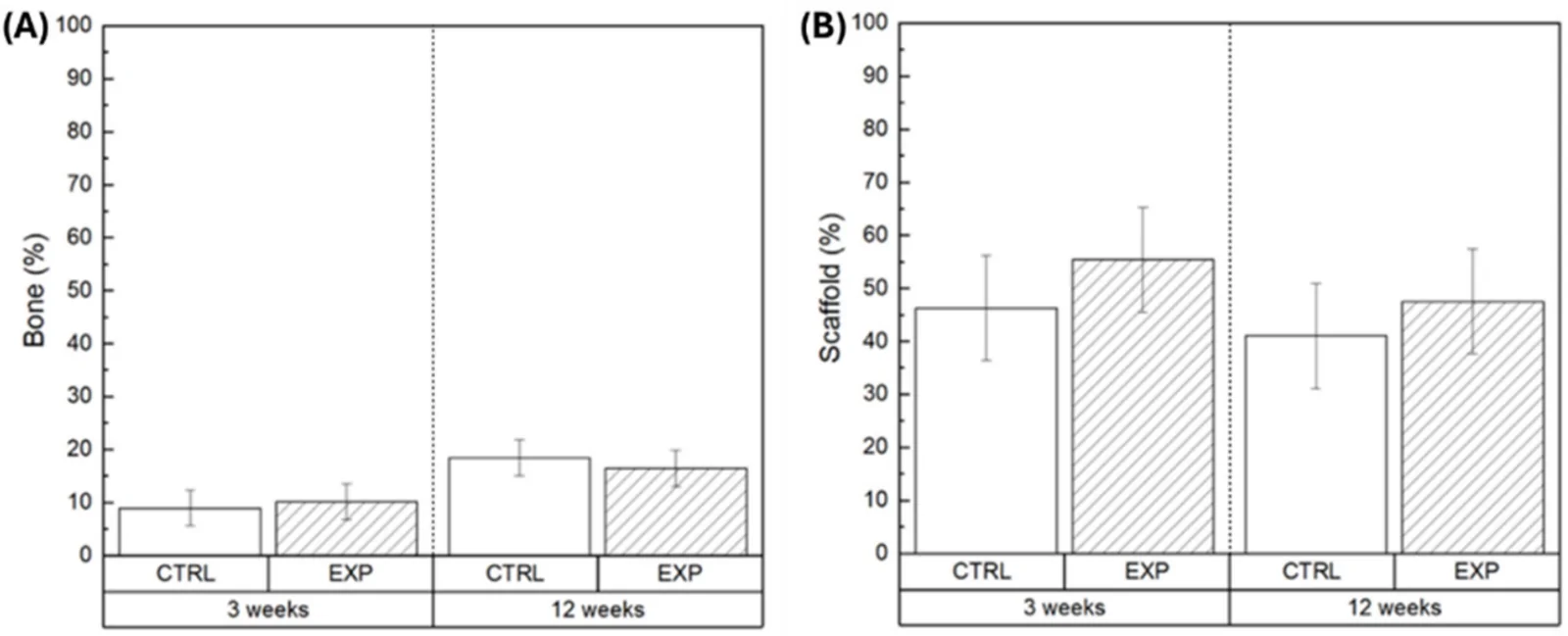
µCT-based volumetric quantification of (**A**) bone (%) and (**B**) scaffold (%) within the induced defects. Values are expressed as means and 95% CI.

Volumetric reconstruction of the defect site at 3 weeks is presented in Figure 3A,B. Both groups evidenced new bone formation at the periphery of the scaffolds owing to the proximity to the osteotomy walls. The scaffolds were observed to be intact with no signs of failure within the implanted site. Adjusting the opacity of the segmented reconstructions revealed minimal bone formation within the inner lattice structures of the scaffold (Figure 3B). In contrast, at 12 weeks, bone growth appeared to have proceeded from the walls of the osteotomy, inwards, towards the center of the scaffolds (Figure 3C,D). The scaffolds (irrespective of the group) promoted bone growth owing to the engineered, lattice-like pore structures (Figure 3D). No other signs of scaffold failure or adverse effects were observed at this extended healing time point. No significant differences in this sample were observed in bone (%) or scaffold (%) between the CTRL and EXP groups at 3 or 12 weeks (*p* > 0.05) (Figure 4A,B).

### 3.3. Histomorphometric Findings

**Figure 5:**
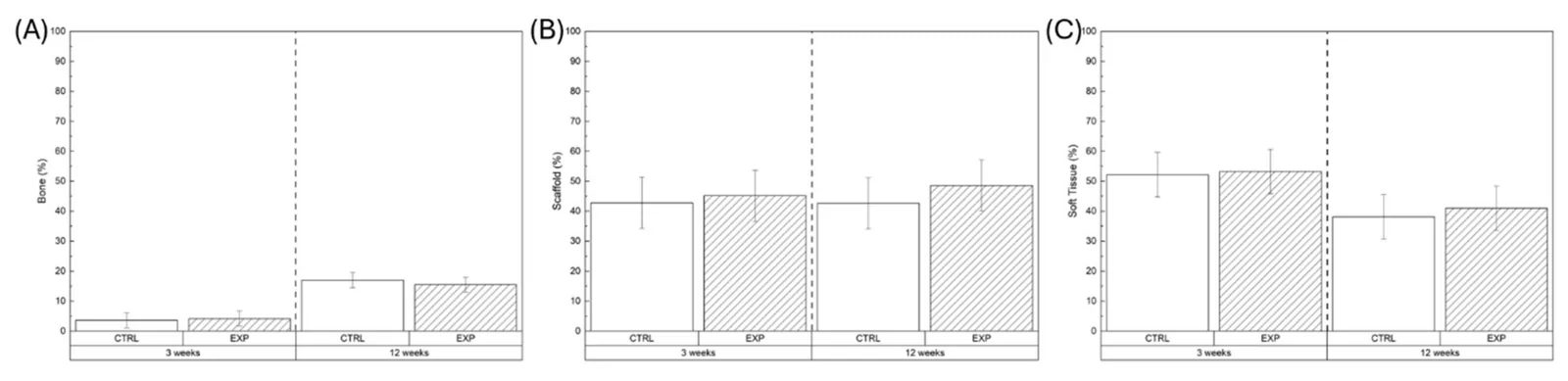
Histomorphometric findings of (**A**) bone (%), (**B**) scaffold (%), and (**C**) soft tissue (%) within the induced defects. Values are expressed as means and 95% CI.

Consistent with volumetric findings, quantitative histological evidence (Figure 5A,B) revealed no statistical differences in bone (%) or scaffold (%) in this sample between the 2 groups at either time point of evaluation (*p* > 0.05). Furthermore, no differences were observed in soft tissue (%) in this sample between CTRL and EXP groups (*p* > 0.05) at 3 or 12 weeks.

## 4. Discussion

The current study aimed to evaluate whether 3D-printed PLGA binder-based self-hardening CaP bone scaffolds are osseoconductive and biocompatible. Ingrowth of newly formed bone was visualized throughout the entire scaffold structure and was not limited to the periphery of the osteotomy walls after 12 weeks of healing. The scaffold lattice structure was filled with woven bone as a result of scaffold pores acting as healing chambers and directing osseoconduction from the periphery, inwards, via an intramembranous-like healing pathway.

A study by Johansson et al. explored the bench-top application of self-hardening scaffolds, where the mechanical brittleness of the scaffolds rendered the overall process of biomechanical fixation to be tedious [28]. To confirm whether the improved mechanical properties could potentially translate to an enhanced fixability of the scaffolds, screwability tests were performed in a challenging, thin reconstruction design in previous work [24,28]. While CTRL scaffolds could be perforated with a drill, the scaffold disintegrated during subsequent fixation [24,28]. In contrast, the EXP scaffolds not only were successfully perforated and fixed to the anatomical model but also stayed intact without visible signs of mechanical failure [24,28]. The current pre-clinical in vivo study did not evidence the aforementioned complications related to surgical placement and mechanical failure in either group evaluated. However, this does not discount the added bench-top mechanical competence of the EXP scaffolds shown in previous work [24].

Pertaining to the synthesis of these scaffolds, a previous study illustrated that the shrinkage associated with the α-TCP setting reaction to CDHA represents minimal distortion-related risks to the overall structure of the scaffold [29]. Of note, specific features obtained by the hydrothermal process, such as the microstructure, nanoporosity, and nanopore size within the 3D-printed structures, were found to be favorable for potential bone regenerative procedures, in addition to the reduced processing times relative to the widely established biomimetic methods [15]. In addition to speeding up hydrolysis, the hydrothermal process has been demonstrated to alter the chemistry and microstructure, which may have an impact on the in vivo performance of the scaffolds [15]. Specifically, in contrast to the plate-like structures produced by biomimetic processing, hydrothermal methods have been found to produce needle-shaped crystals [15]. The needle-shaped morphology has been demonstrated to lower specific surface area, thereby improving the applicability of the hydrothermal process in the synthesis of bone regenerative, tissue engineering bioceramic scaffolds [15]. Hydrothermal incubation may also be more suitable due to its ability to maintain porosity between bundles or crystals to enable appropriate polymer penetration, and owing to its high permeability to allow for the diffusion of protein [15]. In a pre-clinical setting, as shown in the current study, no apparent signs of scaffold fracture were observed in either group at both time points of evaluation, potentially owing to the improved mechanical strength imparted by the hydrothermal process, regardless of the PLGA incorporation.

However, it has been demonstrated that the ester bonds in PLGA undergo chemical hydrolysis as a result of hydrothermal hardening and the sterilization process [30]. Galea et al. observed PLGA to be entangled with the CDHA needle-like crystals on both the filament surface and through the filament cross-section [31]. In previous studies, the EXP samples showed linear elastic behavior until the maximum load was attained, followed by plastic deformation, thereby increasing the total dissipated energy, whereas the CTRL bioceramic scaffolds showed brittle linear elastic behavior until fracture [15,24]. While the mechanical properties of EXP scaffolds in the literature showed a significant improvement over the CTRL counterparts, no significant differences were noted in the in vitro behavior [15,24]—similar to the findings of the current in vivo study. This may be attributed to the presence of PLGA exhibiting a shielding effect on CaP crystals, preventing the cells from making direct contact with CaP, which is the phase that promotes osteogenic differentiation, especially at early healing stages [32].

The homogenous bone healing properties between CTRL and EXP scaffolds in the current study could be attributed to similar physicochemical changes in vivo, which could have influenced the outcome variables, particularly at advanced stages of healing and repair. However, evaluation of bone regeneration starting at the 3-week time point may be perceived as too late to sufficiently elucidate the potential effects of the polymer incorporation. Nonetheless, the promising results at 3 and 12 weeks in the healing cascade suggest that future studies should explore shorter time points to better understand the potential effects of PLGA binders in the bone regeneration process. On the other hand, to ascertain if hard tissue growth will sufficiently replace the scaffold and restore form and function of bone at the defect, longer time points are also warranted in forthcoming studies, including pre-implantation µCT scanning (time-zero or baseline) of the scaffolds within the induced defects to enable direct quantification of resorption. It should also be noted that the 7 mm × 6 mm defect was not independently validated as critical-sized, and the absence of empty-defect or autograft in the current study limits our ability to determine the extent to which the observed bone formation reflects a scaffold-mediated effect versus spontaneous defect healing. However, our previous study in an ovine iliac model revealed limited bone healing 6 weeks post-operatively in 3.8 mm diameter defects prepared using conventional rotary instrumentation, where bone formation was restricted to the periphery of the osteotomies [33]. We hypothesize that the larger (7 mm diameter) defects used in this study may show a lack of spontaneous healing. Future studies incorporating an empty-defect control and/or an autograft comparator will be necessary to definitively isolate the contribution of the scaffold formulations to the observed healing response. Moreover, distinguishing new bone from the CaP scaffold using HU thresholds can be challenging due to overlapping radiopacities, an inherent limitation of µCT. As a single fixed threshold was applied uniformly across all scaffolds in the present study, this potential source of segmentation error was not formally quantified, and future studies should incorporate threshold-sensitivity and intraobserver variability analyses.

Given the number of parameters governing scaffold-mediated bone healing, future work would benefit from complementing in vivo evaluation with computational modeling approaches capable of simulating the healing process across a range of design and material inputs. Recent advances combining computational fluid dynamics with machine learning have demonstrated the feasibility of rapidly predicting scaffold permeability across different architectures [34], and similar data-driven or mechanistic simulation frameworks could, in principle, be extended to model bone ingrowth and remodeling as a function of scaffold architecture, formulation, and defect geometry [34,35]. Such approaches could substantially reduce the experimental burden by prioritizing the most promising design parameters for forthcoming in vivo validation, and represent a promising direction for the continued translation of this material and scaffold system.

## 5. Conclusions

By 12 weeks, hard tissue formation in both scaffold groups showed newly developed vasculature consistent with early-stage woven bone formed through an intramembranous-like pathway. Together with the improved mechanical performance of these formulations relative to pure-ceramic counterparts reported in the literature [15,24], these findings indicate that 3D-printed, PLGA-reinforced, self-hardening CaP scaffolds are both osseoconductive and biocompatible, supporting their potential utility as a bone graft substitute in orthopedic and craniomaxillofacial reconstructive applications. In orthopedic defect repair involving the iliac crest as the donor site, these findings support further evaluation of the EXP formulation as an alternative to autograft harvesting, which could reduce associated donor-site morbidity. In craniomaxillofacial reconstruction, where defect geometries are often irregular and patient-specific, these 3D printing workflows offer a pathway toward customized alloplastic grafts that are matched to individual defect anatomy.

## Data Availability

The data supporting the findings of the study are available from the corresponding author upon reasonable request.

## Supplementary Materials

The following supporting information can be downloaded at: https://www.mdpi.com/article/10.3390/bioengineering13091049/s1, Figure S1: Calibration and phantom data for the µCT scanner (µCT 40, Scanco Medical, Basserdorf, Germany). Table S1: ARRIVE checklist.
