# Supplementary material for: 3D-Printed Poly(Lactic-co-Glycolic Acid) Binder-Based Self-Hardening Calcium Phosphate Bone Scaffolds
Source: Bioengineering (Basel). 2026 Sep 9;13(9):1049. doi: 10.3390/bioengineering13091049 (PMC13603544; doi:10.3390/bioengineering13091049)
Supplement: Supplementary file 1 [file bioengineering-13-01049-s001.zip › C0011873_QC1.pdf]

# QC1 (weekly, density)

**SCANCO** MEDICAL

| | | | |
|---|---|---|---|
| Scanner-ID: | 4263 | Code: | Phantom |
| S-No.: | 1 | Filename: | C0011873 |
| M-No.: | 11997 | Date: | 07−MAR−2024 15:37 |
| E-I: | E2_Imax | Ctrl-File: | QC1 55kVp 200mg HA (optional) |

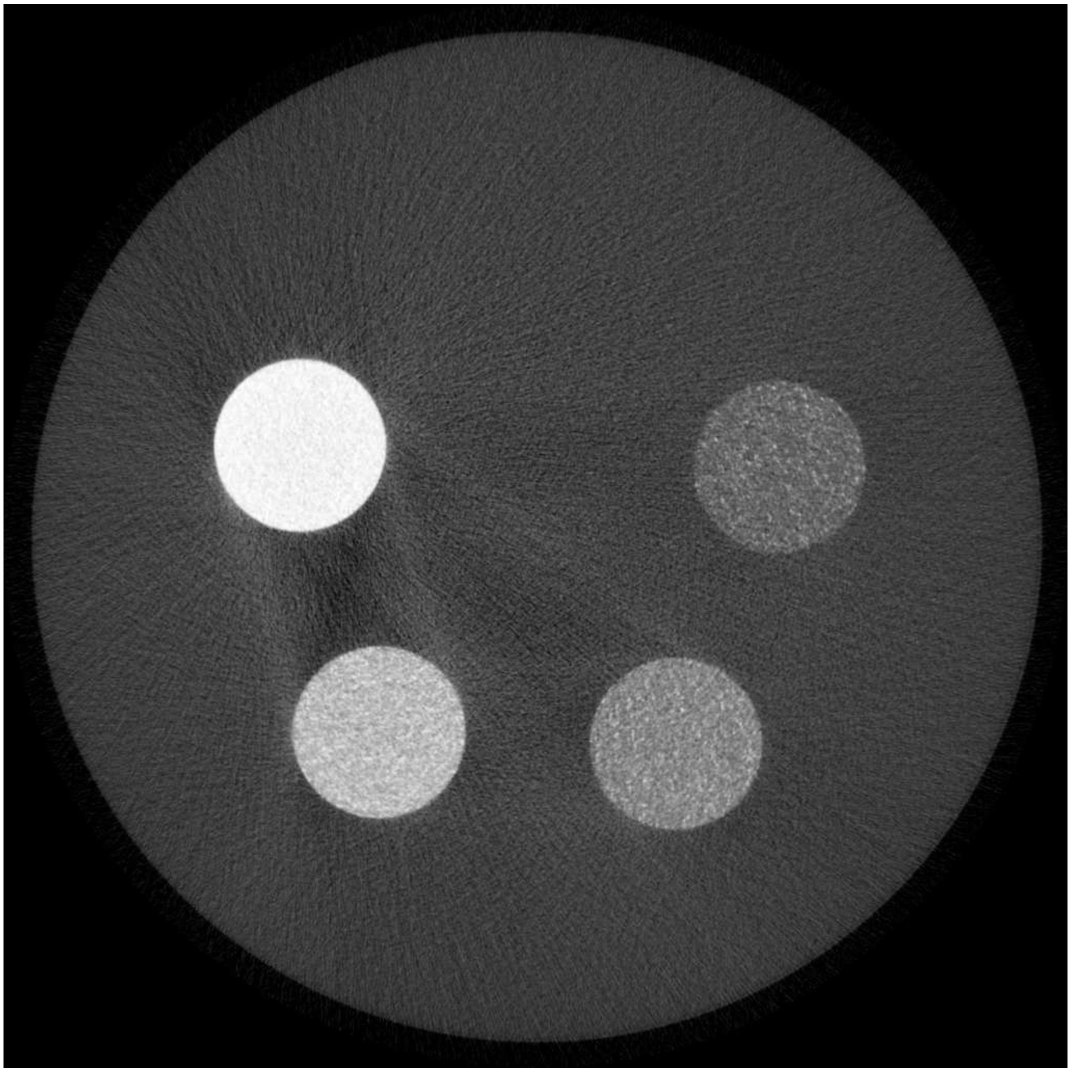

| Cylinder Densities [mg HA/ccm] | | Alu Rod Volume [$mm^3$] |
|---|---|---|
| Mean1 ± SD | −18.2815 ± 26.8331 | |
| Mean2 ± SD | 97.9227 ± 49.2304 | |
| Mean3 ± SD | 208.8184 ± 58.0293 | Rod Volume − |
| Mean4 ± SD | 414.5390 ± 67.2646 | |
| Mean5 ± SD | 780.8221 ± 61.8108 | |
| No of Measured Slices | 103 | Rod Threshold − |

| | | | |
|---|---|---|---|
| Mean | Mean Value of Region in mg HA/ccm | Rod Vol. | Volume of Segmented Aluminium Rod |
| SD | Std.Dev. of Values in Region in mg HA/ccm | | |
| Slope | 297.855 | | |
| Intercept | −188.661 | Printed: | 07−MAR−2024 15:57 |

## 3D Density and Geometry Phantom Evaluation

*μCT*

**SCANCO** MEDICAL
